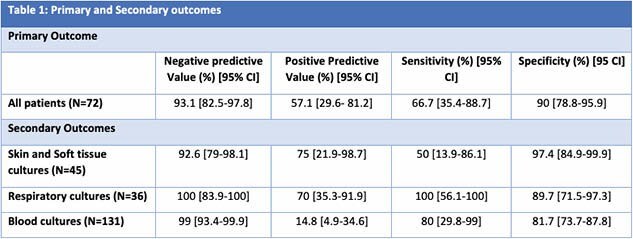# 872 Clinical Utility of Methicillin-resistant Staphylococcus aureus Nares Swabs in Burn Patients

**DOI:** 10.1093/jbcr/iraf019.403

**Published:** 2025-04-01

**Authors:** Jade Montgomery, Rachel Burgoon, Aaron Hamby, Melanie Condeni

**Affiliations:** Medical University of South Carolina; Medical University of South Carolina; Medical University of South Carolina; South Carolina Burn Center at Medical University of South Carolina

## Abstract

**Introduction:**

Methicillin-resistant Staphylococcus aureus (MRSA) is a common pathogen in burn injured patients. Many studies have evaluated the overall utility of MRSA nares swabs for antimicrobial stewardship; however, there is a paucity of data in burn patients. The purpose of this project was to evaluate the diagnostic performance characteristics of MRSA nares in burn patients at an academic medical center.

**Methods:**

This retrospective, single-center chart review included admitted adult burn patients from July 2020 to December 2023. It excluded patients without a MRSA nares swab and a bacterial culture in the same admission, only a urine culture, or a length of stay less than 48 hours. The primary objective was to determine the overall negative predictive value (NPV) of MRSA nares in the burn patient population. Secondary objectives included determining the sensitivity, specificity, positive predictive value (PPV), and the diagnostic accuracy MRSA nares for each specific culture type.

**Results:**

There were 965 patients screened and 72 included. Median (IQR) age was 58 (37, 66) years and most were Caucasian (56.9%) and male (76.4%). The most common burn mechanism was flame (41.7%). Median (IQR) TBSA burn was 18% (5, 31.5) and 23.6% had inhalation injuries. Most patients required surgery (83.3%) and admission to the surgical trauma burn intensive care unit (79.2%). There were 223 cultures evaluated, with blood cultures being most common (58.7%). Fourteen patients had positive MRSA nares and the overall prevalence of MRSA infections was 16.7%. Median (IQR) time from MRSA nares to first culture was 0 days (0,0.6). For the primary outcome, the NPV of MRSA nares was 93.1% [95% CI 82.5-97.8%]. Secondary outcomes are in Table 1.

**Conclusions:**

The high NPV calculated in this study is consistent with previous literature, demonstrating that this test may potentially be used to rule out MRSA infections when negative.

**Applicability of Research to Practice:**

While this retrospective chart review is limited by size, it provides valuable information regarding the utility of MRSA nares in burn patients. Overall, this study showed that MRSA nares may be utilized along with other clinical markers to decrease use of empiric antimicrobials targeting MRSA.

**Funding for the Study:**

There is no external funding for this project.